# Elevation of *MMP1* and *ADAMTS5* mRNA expression in glenohumeral synovia of patients with hypercholesterolemia

**DOI:** 10.1186/s13018-022-02998-6

**Published:** 2022-02-15

**Authors:** Kyoko Muneshige, Kentaro Uchida, Tomonori Kenmoku, Ryo Tazawa, Mitsufumi Nakawaki, Daisuke Ishii, Gen Inoue, Masashi Takaso

**Affiliations:** 1 Department of Orthopedic Surgery, Kitasato University School of Medicine, 1-15-1 Minami-ku, Kitasato, Sagamihara City, Kanagawa 252-0374 Japan; 2 Shonan University of Medical Sciences Research Institute, Nishikubo 500, Chigasaki City, Kanagawa 253-0083 Japan

**Keywords:** Osteoarthritis, Hypercholesterolemia, ADAMTS5, MMP1, Synovium

## Abstract

**Background:**

Epidemiological studies have reported a positive association between hypercholesterolemia and shoulder disease. Previous studies have focused on the effect of hypercholesterolemia on tendinopathy. Moreover, hypercholesterolemia has also been linked to joint pathology in the knee and hand. However, the effect of hyperlipidemia on glenohumeral joint remain unclear. A hypercholesterolemic condition has been reported to alter levels of A Disintegrin and Metalloprotease with Thrombospondin Motifs (ADAMTSs) and matrix metalloproteases (MMPs) in synovium of the knee joint. Here, we evaluated the mRNA expression of ADAMTSs and MMPs in the glenohumeral synovium of patients with and without hypercholesterolemia.

**Methods:**

Study participants were 73 patients who underwent arthroscopic rotator cuff repair for degenerative rotator cuff tears. They were divided into two groups according to total cholesterol (TC) and triglyceride levels. Synovial membrane samples were harvested at the rotator interval during surgery, and mRNA expression levels of the aggrecanases ADAM-TS4 and ADAM-TS5 and MMPs (*MMP-1, 3, 9,* and *13*) were analyzed quantitatively.

**Results:**

*ADAM-TS5* and *MMP1* mRNA levels were significantly higher in the high TC group than in the low TC group (*P* = 0.023 and *P* = 0.025, respectively). In contrast, no significant differences were observed in *ADAMTS4* or *MMPs 3, 9,* and *13* (*ADAMTS4*, *P* = 0.547; *MMP3*, *P* = 0.55; *MMP9*, *P* = 0.521; and *MMP13*, *P* = 0.785).

**Conclusion:**

Hypercholesterolemia may alter MMP1 and ADAMTS5 expression in the synovium of the glenohumeral joint.

## Background

Several systemic factors have been linked with degenerative shoulder diseases, including dyslipidemia, diabetes and obesity [1–3]. Hypercholesterolemia is a systemic metabolic disease characterized by abnormally high levels of cholesterol in the blood which has effects on not only internal organs and cardiovascular tissues but also the musculoskeletal system [4]. Previous studies have focused on the effect of hypercholesterolemia on the rotator cuff [5–7]. However, an evaluation of comorbidities in patients presenting with shoulder osteoarthritis (OA) revealed that 48.7% of patients with primary shoulder OA also suffered from hyperlipidemia [8]. Hypercholesterolemia has also been statistically associated with glenohumeral joint pain but not rotator cuff tendinopathy [9]. Notably, hypercholesterolemia has also been linked with OA in the knee and hand [10, 11]. These observations suggest that hypercholesterolemia affects not only tendons but also joint pathology in the shoulder. Nevertheless, the effect of hyperlipidemia on the glenohumeral joint remains unclear.

The matrix metalloproteinases (MMPs) and A Disintegrin and Metalloproteinase Domain with Thrombospondin motifs (ADAMTS) family play a critical role in the destruction of extracellular matrix in arthritis, rotator cuff disease and other musculoskeletal diseases [12–15]. Synovial tissue is a major source of MMPs and ADAMTS, the expression of which could be affected by dyslipidemia [16, 17]. We previously found that dyslipidemia altered synovial MMP and ADAMTS expression levels in a mouse model of knee OA that exhibited dyslipidemia [16]. Previous studies reported the elevation of MMPs in tendons in patients with tendinopathy [14] and that this elevation was affected by hypercholesterolemia [4]. However, it remains unclear whether dyslipidemia alters MMPs and ADAMTS expression in the synovium of the glenohumeral joint.

Here, we evaluated the mRNA expression of MMP and ADAMTS in the glenohumeral synovium of patients with and without dyslipidemia.

## Methods

### Patients

This study was approved by the Ethics Committee of our institution (Clinical Research Review Board of the Kitasato Institute; reference number KMEO B13-113) and abode by the 1964 Helsinki Declaration and its later amendments or comparable ethical standards. Written informed consent was obtained from all participants.

Synovial membrane samples acquired from the glenohumeral joint, specifically areas of the rotator interval showing redness, were obtained from patients who underwent arthroscopic surgery from November 2017 to October 2020. Patients with RCT were clinically assessed prior to surgery using the Constant score [18]. We graded radiographic OA as normal (no osteophytes), mild (< 3 mm), moderate (3–7 mm), or severe (≥ 7 mm) using the Samilson–Prieto classification [19]. All arthroscopic surgeries to repair a torn rotator cuff were performed by an experienced shoulder surgeon. We excluded patients with a history of rheumatoid arthritis, other collagen diseases, and fractures in the humerus or glenoid. The remaining patients were divided into two groups according to their total cholesterol (TC) level (< 220 mg/dl and ≥ 220 mg/dl), based on the standardized value established by the Japan Atherosclerosis Society (JAS) [20]. Patients were also divided into two groups according to triglyceride (TG) levels (< 150 mg/dl and ≥ 150 mg/dl), also based on the standardized value of the JAS [20].

### qPCR

To extract total RNA, synovial samples were homogenized using a Polytron homogenizer (KINEMATICA AG, Luzerne Strasse, Luzern, Switzerland). After centrifugation (18,000×*g*, 4 °C, 5 min), the supernatants were mixed with an equal volume of 100% ethanol solution, vortexed for 30 s, and then immediately transferred to spin columns (Direct-zol RNA MicroPrep kit; Zymo Research, Irvine, CA, USA) for mRNA isolation. RNA concentration was determined using a DS-11 spectrophotometer (DeNovix, Wilmington, DE, USA). SuperScript™ III Reverse Transcriptase (Thermo Fisher Scientific, Waltham, MA, USA) was used for complementary DNA synthesis. We quantitatively measured the gene expression of ADAMTSs (*ADAMTS4* and *ADAMTS5*) and MMPs (*MMP1, 3, 9,* and *13*) on a real-time PCR detection system (CFX-96; Bio-Rad, Hercules, CA, USA). The PCR primer pair sequences are listed in Table 1. mRNA expression of the *ADAMTS* and *MMP* genes was normalized to that of glyceraldehyde-3-phosphate dehydrogenase (*GAPDH*) using the delta-delta Ct method. When the average expression (genes/*GAPDH*) level in the low HC group was 1, the relative expression was calculated.

**Table 1:** Sequences of primers

Gene	Direction	Primer sequence (5′–3′)	Product size (bp)
*ADAMTS4*	F	AACACTGAGGACTGCCCAAC	159
	R	GGTGAGTTTGCACTGGTCCT	
*ADAMTS5*	F	ATGCACTTCAGCCACCATCA	114
	R	TCGTAGGTCTGTCCTGGGAG	
*MMP1*	F	ACTTACATCGTGTTGCGGCT	164
	R	CGATGGGCTGGACAGGATTT	
*MMP3*	F	GTGGAGTTCCTGACGTTGGT	164
	R	TGGAGTCACCTCTTCCCAGA	
*MMP9*	F	TTTGAGTCCGGTGGACGATG	197
	R	GCTCCTCAAAGACCGAGTCC	
*MMP13*	F	TGACTGAGAGGCTCCGAGAA	111
	R	CATCAGGAACCCCGCATCTT	

### Statistical analysis

Results are expressed as mean ± standard deviation (SD). Statistical significance was determined using the nonparametric Mann–Whitney U test or unpaired *t*-test. Statistical significance was set at *P* < 0.05. All statistical analyses were conducted using the SSPS software v19.0 (IBM, USA).

## Results

### Expression of *ADAMTS* and *MMPS* in patients with low and high TC levels

**Fig. 1:**
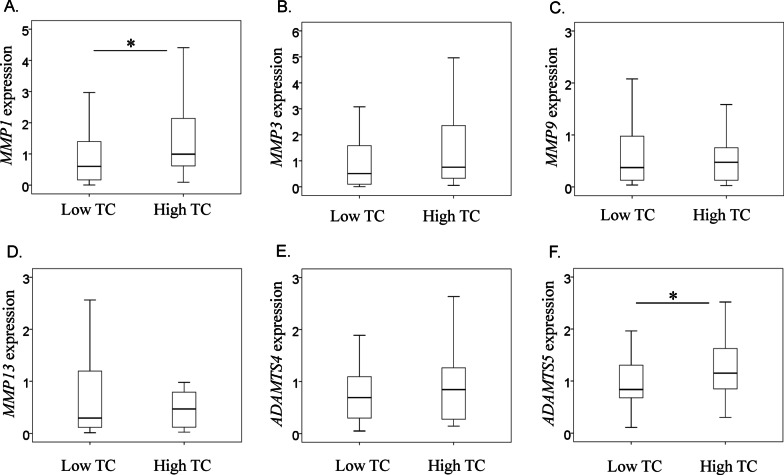
Effect of cholesterol levels on the mRNA expression of MMPs and ADAMTSs mRNA expression levels of **a** *MMP1*, **b** *MMP3*, **c** *MMP9*, **d** *MMP13*, **e** *ADAMTS4*, **f** *ADAMTS5*

The clinical characteristics of patients are summarized in Table 2. Briefly, 42 patients were assigned to the low TC group (27 men and 15 women, aged 65 ± 9 years) and 31 patients were assigned to the high TC group (23 men and 8 women, aged 64 ± 11 years; Table 2). In both groups, no significant differences were observed in patient age at surgery (*P* = 0.505), men/women ratio (*P* = 0.449), body mass index (*P* = 0.539), TG levels (*P* = 0.018), Constant score (*P* = 0.668), or tear size (*P* = 0.465). *ADAMTS5* and *MMP1* levels were significantly higher in the high-TC group than in the low-TC group (*ADAMTS5*, *P* = 0.023; *MMP1,* *P* = 0.025; Fig. 1b, c). In contrast, no significant differences were observed for *ADAMTS4* and *MMP3, 9,* and *13* (*ADAMTS4*, *P* = 0.547; *MMP3*, *P* = 0.55; *MMP9*, *P* = 0.521; *MMP13*, *P* = 0.785; Fig. 1a, d, e, f).

**Table 2:** Clinical characteristics of low and high total cholesterol (TC) groups

	Low TC group	High TC group	*P* value
Age during surgery (years)	65.5 ± 9.2	63.9 ± 10.9	0.647
Number of patients (men/women)	27/15	23/8	0.449
BMI^a^ (kg/m^2^)	25.0 ± 3.4	26.1 ± 4.3	0.539
Total cholesterol level (mg/dl)	182.6 ± 26.1	248.4 ± 27.0	< 0.001
Triglyceride level (mg/dl)	163.6 ± 108.7	243.2 ± 167.8	0.018
Constant score	41.2 ± 17.1^b^	39.3 ± 17.8^c^	0.668
Tear sizes of patients (partial/small or middle/large or massive)	8/17/17	5/9/17	0.465
Samilson–Prieto classification of patients (normal/mild/moderate/severe)	4/15/22/1	6/11/14/0	0.535

^a^Body mass index

^b^Information missing for seven patients

^c^Information missing for four patients

### Expression of *ADAMTS *and *MMPs* in patients with low and high TG levels

**Fig. 2:**
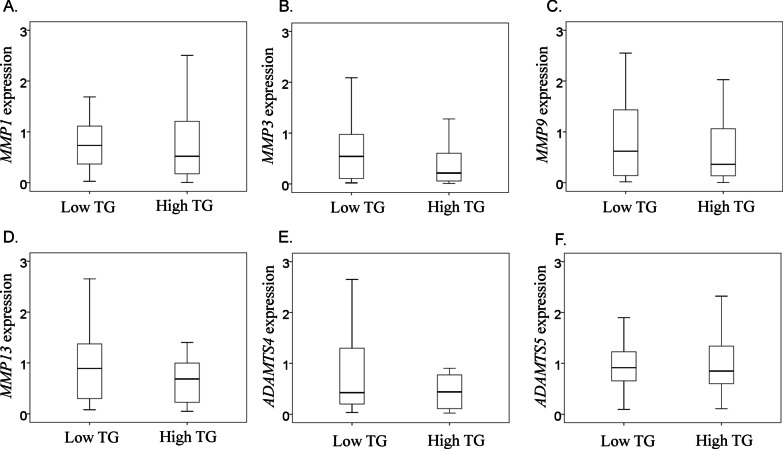
Effect of triglyceride levels on the mRNA expression of MMPs and ADAMTSs **a** mRNA expression levels of *MMP1*, **b** *MMP3*, **c** *MMP9*, **d** *MMP13*, **e** *ADAMTS4*, and **f** *ADAMTS5*

According to the criteria, 33 patients were assigned to the low-TG group (16 men and 17 women, aged 65 ± 8 years) and 40 patients were assigned to the high-TG group (34 men and 6 women, aged 64 ± 11 years; Table 3). No significant differences between groups were found in the average age of patients at surgery (*P* = 0.846), body mass index (*P* = 0.094), total cholesterol level (*P* = 0.202), Constant score (*P* = 0.549), or tear size (*P* = 0.516). However, significant differences were observed in the men/women ratio (*P* = 0.001). No significant differences in mRNA expression levels were detected between the two groups (Fig. 2).

**Table 3:** Clinical characteristics of low and high total triglyceride (TG) groups

	Low TG group	High TG group	*P*-value
Age during surgery (years)	65.5 ± 8.3	64.2 ± 11.2	0.846
Number of patients (men/women)	16/17	34/6	0.001
BMI^a^ (kg/m^2^)	24.6 ± 5.5	26.2 ± 4.0	0.094
Constant score	41.7 ± 17.2^b^	38.9 ± 17.6^c^	0.549
Total cholesterol level (mg/dl)	202.7 ± 38.4	217.1 ± 44.1	0.202
Triglyceride level (mg/dl)	98.7 ± 33.3	278.4 ± 145.1	< 0.001
Tear sizes of patients (partial/small or middle/large or massive)	4/13/16	9/13/18	0.501
Samilson–Prieto classification of patients (normal/mild/moderate/severe)	5/14/14/0	5/12/22/1	0.516

^a^Body mass index

^b^Information missing for three patients

^c^Information missing for eleven patients

## Discussion

In this study, *ADAMTS5* and *MMP1* expression in the synovial membrane of the glenohumeral joint was found to be significantly higher in patients with high TC levels than in those with low TC levels. In contrast, no significant differences were observed between the two TG groups. Therefore, hypercholesterolemia altered synovial *MMP1* and *ADAMTS5* levels in the glenohumeral joint.

MMP-1 is primarily produced by the synovial cells that line the joints [12]. MMP-1 has the unique ability to cleave the triple helix of collagen. Cleavage allows the chains to unwind, which makes them susceptible to further degradation by other MMPs [21]. A relationship between *MMP1* expression and cholesterol levels has been identified in both in vitro and in vivo animal studies [22–24]. Cholesterol crystals stimulate *MMP1* mRNA expression in human macrophages in vitro [22]. Oxidized LDL promotes MMP1 production in cultured synoviocytes derived from patients with rheumatoid arthritis [23]. Hypercholesterolemic rabbits exhibited increased MMP1 levels in the aorta [24]. In this study, *MMP1* expression in the glenohumeral synovia was confirmed to significantly differ between patients with high and low TC levels, although this was not confirmed between patients with high and low TG levels. Rotator cuff MMP1 levels in synovial fluid are higher in patients with rotator tears than in healthy controls [25]. MMP1 levels in RCT patients with a massive full thickness tear were significantly higher than in patients with a partial-thickness tear and a non-massive full-thickness tear [26]. In our study, tear size did not differ tear between the high TC and low TC groups, suggesting the possibility that hypercholesterolemia increases MMP1 levels in the glenohumeral joint.

ADAMTS5 has emerged as a principal mediator of aggrecan loss in OA [27, 28]. *ADAMTS-5* knockout mice were reportedly protected from synovitis and joint destruction [29, 30]. Some studies have focused on the relationship between ADAMTS5 and cholesterol levels. ADAMTS5 was elevated in the nucleus pulposus cells of APO-E knockout rabbits [31]. Intra-articular anti-ADAMTS5 antibody slowed down the progression of OA in a dose-dependent manner in a murine OA model with hypercholesterolemia [32]. We found that patients with hypercholesterolemia had higher expression levels of synovial *ADAMTS5*. A previous study reported that cartilage obtained from patients with shoulder OA had higher *ADAMTS5* mRNA expression than cartilage obtained from non-OA patients [33]. Some of our present patients had OA. However, no significant difference was found in OA grade in the high and low TC groups. Therefore, our findings suggest that hypercholesterolemia might also affect *ADAMTS5* expression in the glenohumeral synovium.

Several studies have reported a possible association between knee OA and TG levels, albeit that none of these associations reached statistical significance [10, 34, 35]. In our study, expression of *MMPs* and *ADAMTSs* did not differ between patients with low and high TG levels. Corroborating previous findings [10, 34, 35], our study suggests that the presence of hypertriglyceridemia has less influence on the glenohumeral joint.

Hypercholesterolemia leads to structural, inflammatory and mechanical changes in tendons, which predispose hypercholesterolemic patients to a greater risk of tendon pathology [4]. Shoulder arthritis can occur in patients with RCT, and in severe cases, the cartilage degeneration may be related to extra mechanical loading resulting from the rotator insufficiency [36]. Given that impaired mechanical loading increases MMP1 and ADAMTS5 in synovial cells [37, 38], our results might partly reflect the upregulation of mechanical stress factors due to tendinopathy. Further investigation using non-RCT patients may help reveal the direct link between synovial pathology and hypercholesterolemia.

Several limitations of this study warrant mention. First, the major limitation is that the only measure used was PCR. Protein profiling studies such as western blot and immunohistochemical analysis are needed to validate our gene expression profile results. Second, we did not include a healthy control population, although this would have been preferable as an ideal control group. Finally, any directly link in the etiology of OA with MMP1 and ADAMTS5 remains unclear.

## Conclusions

Hypercholesterolemia may alter MMP1 and ADAMTS5 expression in synovium of the glenohumeral joint.

## Data Availability

Datasets supporting the conclusions of this article are included within the article. The raw data can be requested from the corresponding author.

## Abbreviations

OA: Osteoarthritis
ADAMTSs: A Disintegrin and Metalloprotease with Thrombospondin Motifs
MMPs: Matrix metalloproteases
RCTs: Rotator cuff tears
TC: Total cholesterol
TG: Triglyceride
JAS: Japan Atherosclerosis Society
SD: Standard deviation
GAPDH: Glyceraldehyde-3-phosphate dehydrogenase
